# Comparative evaluation of VoxelMorpand conventional deformable image registration algorithms for thoracic 4D‐CT in radiotherapy

**DOI:** 10.1002/acm2.70816

**Published:** 2026-09-23

**Authors:** Mizuha Sakai, Megumi Nakao, Hideaki Hirashima, Masahiro Yoneyama, Noriko Kishi, Takashi Mizowaki, Mitsuhiro Nakamura

**Affiliations:** ^1^ Department of Advanced Medical Physics Graduate School of Medicine Kyoto University Kyoto Kyoto Japan; ^2^ Department of Biomedical Engineering and Intelligence Graduate School of Medicine Kyoto University Kyoto Kyoto Japan; ^3^ Department of Radiation Oncology and Image‐Applied Therapy Graduate School of Medicine Kyoto University Kyoto Kyoto Japan

**Keywords:** deep‐learning based DIR, Deformable image registration, lung cancer, Thoracic 4D‐CT, VoxelMorph

## Abstract

**Background:**

Deformable image registration (DIR) is essential for thoracic four‐dimensional computed tomography (4D‐CT)‐based radiotherapy applications. Recently, deep learning‐based DIR methods such as VoxelMorph have been proposed; however, their performance relative to clinically used DIR algorithms remains unclear.

**Purpose:**

This study aimed to evaluate the DIR accuracy of VoxelMorph for thoracic 4D‐CT and to compare it with conventional clinical and research‐oriented DIR methods.

**Materials and methods:**

Thoracic 4D‐CT data from 64 lung cancer patients were retrospectively analyzed. End‐inhalation and end‐exhalation phase images were used for DIR. VoxelMorph was trained using 50 cases, with 4 for validation and 10 for testing. DIR performance on the test dataset was compared with Demons (SimpleITK), modified Demons (Eclipse), and ANACONDA (RayStation). Accuracy was evaluated by mean absolute error (MAE) of CT values computed within the body region, whereas Dice similarity coefficient (DSC) and 95th percentile of Hausdorff distance (HD95) were evaluated within the lung label.

**Results:**

The median MAE decreased from 67.56 HU before DIR to 52.32 HU with modified Demons, 37.39 HU with Demons, 36.18 HU with ANACONDA, and 36.83 HU with VoxelMorph. The median DSC increased from 0.91 to 0.95 for modified Demons, 0.97 for Demons and ANACONDA, and 0.98 for VoxelMorph. The median HD95 was 3.0 mm for modified Demons, 3.1 mm for Demons, 2.0 mm for ANACONDA, and 2.5 mm for VoxelMorph. Overall, VoxelMorph demonstrated competitive accuracy, significantly outperforming modified Demons (adjusted *p <* 0.05), while showing smaller inter‐case variability and markedly reduced processing time.

**Conclusions:**

VoxelMorph demonstrated DIR performance comparable to that of clinically used algorithms for thoracic 4D‐CT, with high overlap accuracy, relatively low inter‐case variability and shorter processing times under the evaluated implementation conditions. These findings suggest its potential as research‐oriented DIR framework, although further validation under standardized conditions is required before routine clinical implementation.

## 1 INTRODUCTION

Four‐dimensional computed tomography (4D‐CT), generated by synchronizing three‐dimensional CT (3D‐CT) acquisition with respiratory signals, enables the assessment of respiratory‐induced anatomical motion and has become essential for accurate radiotherapy of tumors affected by respiratory motion, such as lung cancer. It is widely used for applications including dose accumulation,^1^ contour propagation,^2^ and lung ventilation analysis.^3^ These applications require deformable image registration (DIR),^2^ which generates a displacement vector field (DVF) to establish voxel‐wise correspondence between moving and fixed images. Registration accuracy is critical in radiotherapy because it directly affects treatment safety and the reliability of dose evaluation. This is particularly important for lung imaging, where respiratory motion induces complex and highly nonlinear anatomical deformations, making accurate DIR essential for reliable image‐based clinical analysis.

Although numerous DIR algorithms have been proposed, conventional methods generally rely on iterative optimization of transformation parameters to maximize image similarity between moving and fixed images. Consequently, these approaches impose a substantial computational burden, resulting in prolonged processing times.^4^ Furthermore, the optimization must be performed independently for each image pair, which represents a major limitation of conventional DIR techniques.^5^

In recent years, deep learning (DL)‐based DIR models have been developed to address these limitations, with VoxelMorph^6^ being one of the most widely adopted and extensively validated frameworks. VoxelMorph estimates a DVF, which is subsequently applied to the moving image, following a workflow conceptually similar to conventional DIR methods. Once trained, VoxelMorph performs DIR by simply providing an image pair to the model, eliminating the need for iterative optimization for each individual case. Consequently, VoxelMorph offers substantially improved computational efficiency and does not require parameter tuning during inference, and has therefore been widely adopted in DIR‐related research.^7^ Despite these advantages, DL‐based DIR methods have not yet been widely implemented in clinical practice, as their stability and robustness relative to conventional DIR approaches have not been fully established.^8^ Several recent studies have evaluated VoxelMorph using thoracic 4D‐CT datasets and demonstrated registration performance comparable to conventional research‐oriented DIR algorithms.^9^, ^10^, ^11^ Furthermore, Lei et al. compared a DL‐based DIR framework with the commercially available VelocityAI software (Varian Medical Systems, Palo Alto, CA, USA) and demonstrated promising results for abdominal 4D‐CT registration.^12^ However, few studies have directly compared VoxelMorph, a widely used benchmark framework for DL‐based DIR, with commercial DIR algorithms implemented in contemporary radiotherapy treatment planning systems, such as Eclipse (Varian Medical Systems) and RayStation (RaySearch Laboratories AB, Stockholm, Sweden). Consequently, the relative performance and practical clinical positioning of VoxelMorph with respect to routinely used clinical DIR frameworks have not yet been fully established.

The aim of this study was to evaluate the performance of VoxelMorph for DIR between end‐inhalation and end‐exhalation 3D‐CT images derived from thoracic 4D‐CT and to directly compare its performance with that of commercially available DIR algorithms used in clinical radiotherapy treatment planning systems. A research‐oriented DIR algorithm was also included as a reference. Through this comparison, we sought to clarify the current clinical positioning of VoxelMorph and its potential role in radiotherapy applications.

## 2 MATERIAL AND METHODS

### 2.1 Patients

The use of the patient data in this study was approved by the institution ethics review board (R1446‐3).

This retrospective study initially included thoracic 4D‐CT data from 65 patients with lung cancer who underwent radiotherapy at Kyoto University Hospital. Each 4D‐CT dataset comprised ten 3D‐CT images corresponding to distinct respiratory phases within a single breathing cycle (*T =* 00, 10, …, 90). For DIR, the end‐inhalation phase (*T =* 00) and the end‐exhalation phase (*T =* 50) were selected, as they exhibit the largest respiratory displacement. Each 3D‐CT image consisted of 136 to 257 slices with an in‐plane matrix of 512 × 512 pixels. The voxel sizes were 0.97 × 0.97 × 2.0 mm^3^, 0.96 × 0.96 × 2.0 mm^3^, or 1.71 × 1.71 × 2.5 mm^3^.

The 65 cases were randomly divided into 50 cases for training, 5 for validation, and 10 for testing. One case in the validation cohort was subsequently excluded because the scan range did not cover the entire lungs and therefore did not satisfy the imaging requirements for whole‐lung evaluation. This exclusion was based solely on scan coverage and was independent of registration performance. Consequently, 64 cases (50 training, 4 validation, and 10 test cases) were included in the final analysis. The mean respiratory motion range, defined as the superior–inferior displacement between end‐inhalation and end‐exhalation on the central coronal slice, was 16.8 ± 9.7 mm, 15.0 ± 7.5 mm, and 19.2 ± 13.9 mm for the training, validation, and test datasets, respectively.

### 2.2 Preprocessing

The following preprocessing steps were applied to prepare the dataset for VoxelMorph. First, a lung mask was generated from the lung contour at *T =* 00, corresponding to the phase with the maximal lung volume. Using this lung mask, the image volumes were cropped along the cranio‐caudal direction by extending the most cranial and caudal lung boundaries by 20 mm in each direction. The cropped volumes were then resampled to an isotropic voxel size of 1 × 1 × 1 mm^3^. Zero padding was applied to center the volumes and adjust their dimensions to 512^3^ voxels. Due to memory limitations during VoxelMorph training, the volumes were further downsampled to 128^3^ voxels. Finally, CT values were clipped to the range of ‐1000 to 1000 HU and normalized to [0, 1]. Through these preprocessing steps, the imaging range, spatial resolution, and volume dimensions were standardized across all cases.

### 2.3 VoxelMorph‐based deformable image registration

**FIGURE 1:**
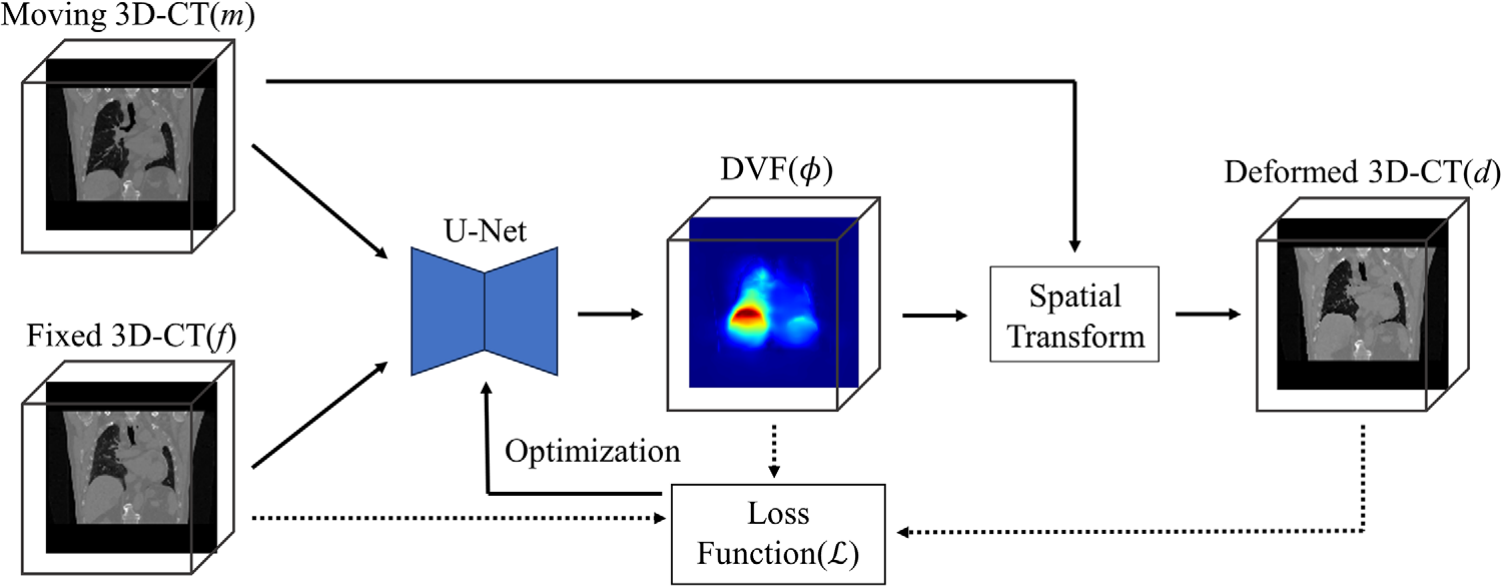
Architecture of VoxelMorph. VoxelMorph takes a pair of moving and fixed images as input and outputs a displacement vector field (DVF). The deformed image is subsequently generated by applying the DVF to the moving image.

The architecture of VoxelMorph used in this study is illustrated in Figure 1. The 3D‐CT image at *T =* 00 was designated as the moving image *m*, and that at *T =* 50 as the fixed image *f*. The images were input as a pair to a U‐Net‐based network, which estimates a DVF, denoted as ϕ. The DVF represents the voxel‐wise displacement from *T =* 00 to *T =* 50. Because the input image size was 128^3^ voxels, the resulting DVF had the same spatial dimensions, with three channels corresponding to the three spatial directions. The estimated DVF was subsequently applied to the moving image using a Spatial Transformer Network^13^ to generate the deformed image *d*. The deformation process is defined by the following equation (1).

(1)

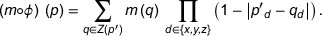




The deformed position $p^{\prime }$ corresponds to the voxel location obtained by applying the DVF to voxel p. Here, $Z(p^{\prime })$ denotes the eight voxels forming the 2×2×2 neighborhood surrounding $p^{\prime }$, which are used for trilinear interpolation, and *d* represents the spatial dimensionality. The loss function is defined as the sum of a mean squared error (MSE) and a regularization term imposed on the DVF, as expressed in the following equation (2).

(2) $$\text{Loss}\left(L\right)=\frac{1}{\left|\Omega \right|}\sum_{p\in \Omega }{\left[f\left(p\right)-\left[m\circ \phi \right]\left(p\right)\right]}^{2}+\lambda \sum_{p\in \Omega }{∥\nabla u\left(p\right)∥}^{2}.$$

Here, Ω denotes the spatial domain of the image, and ∇u(p) represents the spatial gradient of the displacement vector at voxel p. The parameter λ controls the relative contribution of the regularization term. During inference, the moving and fixed images are input to the trained U‐Net to directly estimate the DVF and generate the corresponding deformed image, without any case‐specific optimization or iterative parameter updating.

### 2.4 Experiments

Comparative experiments were conducted to evaluate the DIR accuracy of VoxelMorph against other DIR algorithms. For the VoxelMorph‐based experiments, the dataset described in Section 2.2 was used. The network was trained for 300 epochs with a batch size of 4. The model at epoch 266, which achieved the lowest validation loss, was used for inference. In addition, the regularization coefficient λ in the loss function was varied among 0.005, 0.01, 0.05, and 0.1 to examine its influence on DIR performance. Registration accuracy and deformation regularity were evaluated using the mean absolute error (MAE) and the number of voxels with negative Jacobian determinants $|J_{\phi }\left(p\right)|$, which is defined by equation (3).

(3) $$\left|J_{\phi }\left(p\right)\right|=\left|\begin{array}{ccc} \frac{\partial x^{\prime }}{\partial x} & \frac{\partial x^{\prime }}{\partial y} & \frac{\partial x^{\prime }}{\partial z}\\ \frac{\partial y^{\prime }}{\partial x} & \frac{\partial y^{\prime }}{\partial y} & \frac{\partial y^{\prime }}{\partial z}\\ \frac{\partial z^{\prime }}{\partial x} & \frac{\partial z^{\prime }}{\partial y} & \frac{\partial z^{\prime }}{\partial z} \end{array}\right|\;.$$

Here, (x,y,z) denotes the coordinates of a point before deformation, and $\left(x^{\prime },y^{\prime },z^{\prime }\right)$ the corresponding coordinates after deformation. The Jacobian determinant provides a quantitative measure of local volume change induced by DIR and is computed from the estimated DVF. Values of $|J_{\phi }\left(p\right)|$> 1 indicate local volume expansion, $|J_{\phi }\left(p\right)|$= 1 indicates volume preservation, and 0 <$\;|J_{\phi }\left(p\right)|$< 1 indicates local volume contraction, whereas $|J_{\phi }\left(p\right)|$≤0 indicates image folding and thus non‐physical deformation. Such non‐physical deformations violate anatomical topology, and are therefore unacceptable for clinical applications. Accordingly, DVF regularity was evaluated by counting the number of voxels with $|J_{\phi }\left(p\right)|$≤0.

The regularization coefficient λ was optimized using the validation cohort. The selected value was then fixed and applied to all subsequent experiments on the test cohort.

#### 2.4.1 Comparison with other deformable image registration algorithms

The Demons algorithm implemented in SimpleITK (version 2.5.2; Insight Software Consortium, Seattle, WA, USA) is an intensity‐based DIR method based on the optical‐flow formulation proposed by Thirion and incorporates displacement‐field regularization.^14^ Registration was performed for 500 iterations, with Gaussian smoothing applied to both the update and DVF using a standard deviation of 1.0. The modified Demons algorithm implemented in Eclipse is also an intensity‐based iterative DIR method derived from the Demons framework and uses a multi‐resolution optimization scheme to progressively refine the deformation.^15^ Because the detailed modifications to the Demons formulation and optimization procedure in the commercial implementation are proprietary, they are not fully disclosed. The ANACONDA algorithm implemented in RayStation formulates DIR as a nonlinear optimization problem that combines image‐intensity similarity with regularization of the deformation field and can additionally incorporate anatomical information from contoured structures.^16^ In this study, anatomical information was not incorporated. The modified Demons and ANACONDA algorithms were performed using the default clinical settings provided by each treatment planning system, without additional parameter tuning. Reconstruction artifacts present in the original CT images were not specifically corrected or masked before registration and were retained through the method‐specific preprocessing applied to all four DIR methods.

Due to computational memory limitations, both VoxelMorph and the Demons algorithm were applied to images resized to 128^3^ voxels, resulting in DVFs with the same spatial dimensions. The grid spacing of these DVFs was 4.0 × 4.0 × 4.0 mm^3^. In contrast, the modified Demons and ANACONDA algorithms were implemented within commercial treatment planning systems, which process images at their original spatial resolution (512 × 512 × *N* slices, where *N* denotes the number of axial slices). Consequently, the corresponding DVFs had spatial dimensions of 512 ×512 × *N*. The grid spacing was 1.95 × 1.95 × 1.95 mm^3^ or 2.34 × 2.34 × 2.5 mm^3^ for the modified Demons algorithm, and 2.5 × 2.5 × 2.5 mm^3^ for ANACONDA.

To facilitate comparison across the four DIR methods, the generated DVFs were resampled to a common image grid for evaluation. Specifically, the DVFs generated by VoxelMorph and the Demons algorithm were upsampled using linear interpolation to match the spatial dimensions (512 × 512 × *N* voxels) and grid spacing (1.0 × 1.0 × 1.0 mm^3^) used for evaluation. Similarly, the DVFs generated by the modified Demons and ANACONDA algorithms were resampled to a grid spacing of 1.0 × 1.0 × 1.0 mm^3^ using linear interpolation. Subsequently, the post‐processing procedures described in Section 2.2, excluding the resizing step and HU scaling, were applied to restore imaging coverage and spatial resolution consistent with the original 3D‐CT images. Finally, these standardized DVFs from all four methods were applied to the moving images to generate deformed images with a size of 512^3^ voxels, which were used for comparative evaluation.

#### 2.4.2 Quantitative evaluation

For quantitative evaluation, the MAE of CT values, the Dice similarity coefficient (DSC), and 95^th^ percentile of Hausdorff distance (HD95) were used. No artifact‐specific exclusion was applied during the quantitative evaluation. The MAE was defined as the mean absolute voxel‐wise difference in CT values between the fixed and deformed image, with lower values indicating better agreement. Prior to MAE calculation, CT values of both the fixed and deformed images obtained from all DIR methods were clipped to the range of ‐1000 to 1000 HU, consistent with the preprocessing applied during VoxelMorph training. The MAE was then calculated directly in HU units without intensity normalization within the body region, defined as voxels with CT values ≥ ‐300 HU. The DSC was computed for both lungs, with values closer to 1 indicating better overlap between lung contours. The lung label of deformed image *L_d_* was obtained by applying the estimated DVF to the lung label of the moving image, whereas the lung label of the fixed image *L_f_* was derived directly from its contour information. The overlap between *L_d_* and *L_f_* was used to assess contour agreement. The DSC is defined by the following equation (4).

(4) $$\mathrm{DSC}\;\left(L_{f},\;L_{d}\right)=\frac{2\left|L_{f}\cap L_{d}\right|}{\left|L_{f}\right|+\left|L_{d}\right|}\;.$$

The HD95 was calculated using lung contour information. HD defined by the following equation (5).

(5) $$\mathrm{HD}\;\left(A,\;B\right)=\mathrm{ma}x_{a\in A}\;\left\{\mathrm{mi}n_{b\in B}\left\{d\left(a,\;b\right)\right\}\right\}.$$

A and B denote the sets of points on the lung contour in the fixed and deformed images, respectively. For arbitrary points a∈A and b∈B, d(a,b) denotes the distance between them.

Statistical comparisons of MAE, DSC, and HD95 among the four methods were performed using the Friedman test, with the significance level of 0.05. When a significant overall difference was detected, post hoc pairwise comparisons were conducted using the Wilcoxon signed‐rank test with Holm correction to account for multiple comparisons. In addition, DIR processing time was recorded for each method for reference.

#### 2.4.3 Experimental environment

The experiments using VoxelMorph and Demons were conducted on a workstation equipped with an Intel® Core ™ i9‐10980XE processor (3.00 GHz), 256 GB of RAM, and NVIDIA RTX A5000 GPU with 24 GB of memory. The software environment consisted of CUDA version 11.2 and Python version 3.9.21. VoxelMorph was implemented using the TensorFlow framework. The modified Demons experiments were performed on a system equipped with an Intel Xeon Silver 4110 processor (2.10 GHz), 32 GB of RAM, and an NVIDIA T400 GPU with 4 GB of memory. The ANACONDA experiments were conducted on a system equipped with an Intel 64 Family 6 Model 183 processor (32 cores), 63.69 GB of RAM, and an NVIDIA RTX 5000 Ada Generation Laptop GPU with 14.99 GB of memory.

VoxelMorph, modified Demons, and ANACONDA performed DIR using GPU acceleration, whereas the Demons algorithm was executed using CPU‐based computation.

The libraries used for metrics computation and statistical analysis were as follows: NumPy version 2.3.3 was used to compute MAE and DSC, SciPy version 1.16.2 was used to calculate HD95 and to perform the Wilcoxon signed‐rank test, and Statsmodels version 0.14.5 was used for multiple comparison correction.

## 3 RESULTS

**FIGURE 2:**
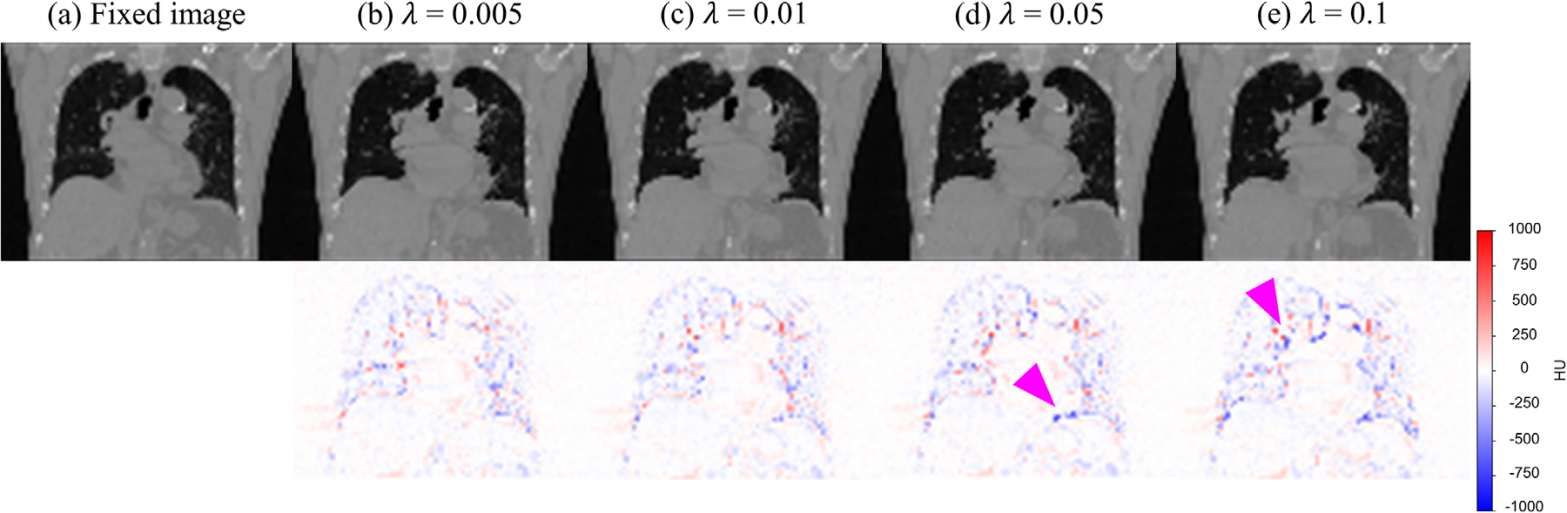
Fixed image, deformed images obtained with different values of the regularization coefficient λ, and difference images between deformed and fixed images. (a) Fixed image, (b) λ = 0.005, (c) λ = 0.01, (d) λ = 0.05, and (e) λ = 0.1. The degradation in deformation accuracy for fine anatomical structures is observed with increasing λ (purple arrows).

Table 1 summarizes the effects of varying the regularization coefficient λ on DIR performance. Small λ values were associated with reduced MAE but increased numbers of voxels exhibiting negative Jacobian determinants $|J_{\phi }\left(p\right)|$. Representative deformed images for each λ are shown in Figure 2. Visual assessment revealed progressive degradation in deformation accuracy for fine anatomical structures with increasing λ. Considering both visual and quantitative evaluations, λ = 0.01 was determined to provide an optimal balance between registration accuracy and deformation regularity and was therefore adopted for subsequent experiments.

**TABLE 1:** Quantitative evaluation of deformable image registration and displacement vector field (DVF) with different regularization coefficient *λ* in 4 validation cases. The median mean absolute error (MAE) between the deformed and fixed images, and the total number of voxels with negative Jacobian determinants in the resulting DVF, when varying the regularization coefficient *λ*. The total number of voxels is 128^3^ voxels × 4 cases.

λ	MAE [HU]	Number of voxels with negative $|J_{\phi }\left(p\right)|$ (percentage)
0.005	22.07	130 (0.0015%)
0.01	23.27	18 (0.0002%)
0.05	25.68	0 (0.0000%)
0.1	27.09	0(0.0000%)

**FIGURE 3:**
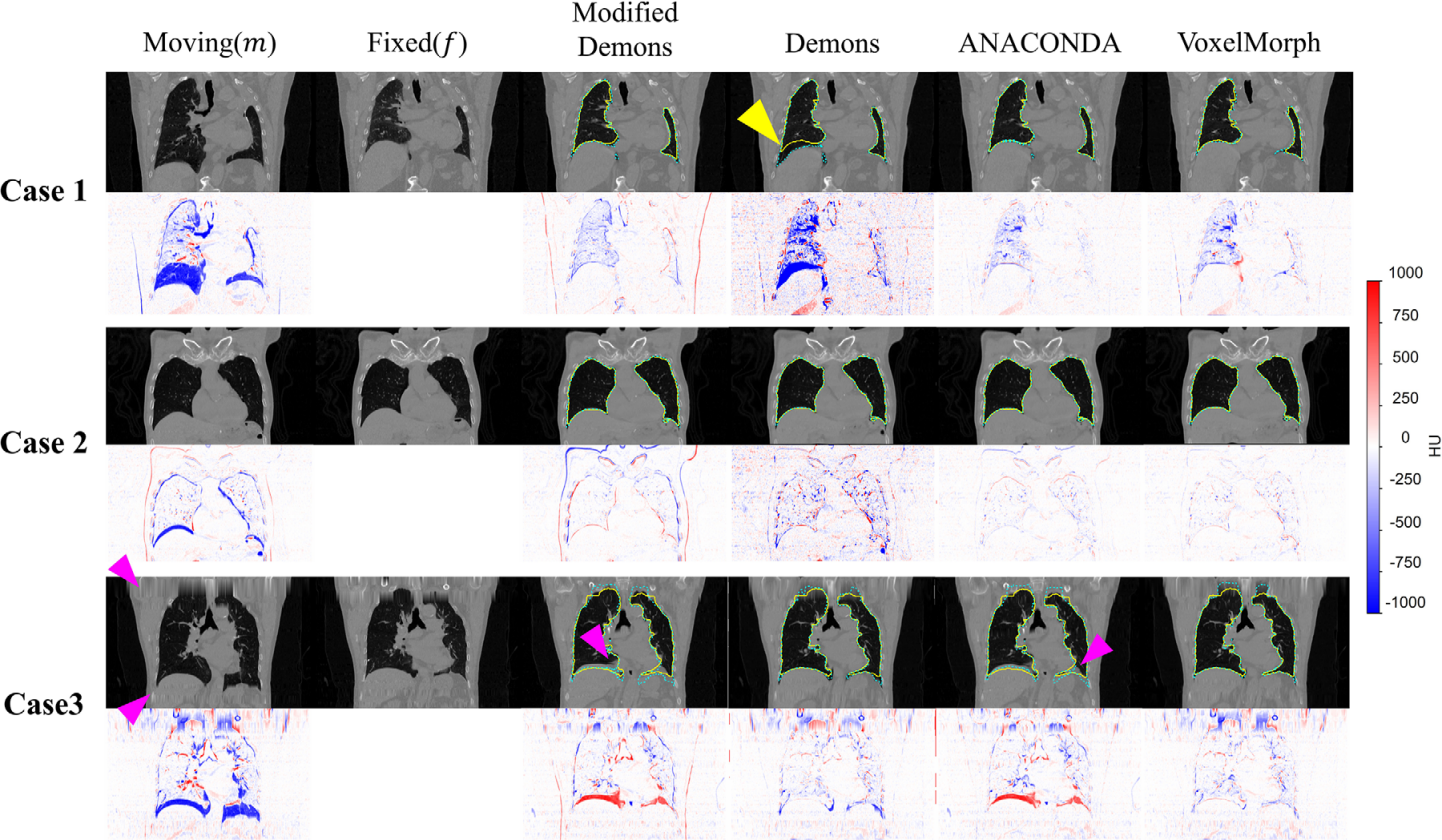
Qualitative comparison of the four DIR methods. For each case, the moving image, fixed image, and the deformed images generated by modified Demons, Demons, ANACONDA, and VoxelMorph are shown, together with the corresponding difference images between the fixed and deformed images. The fixed lung contours (yellow solid line) and propagated lung contours (cyan dashed line) are overlaid on the deformed images to facilitate visual assessment of contour agreement. Case 1 shows large respiratory motion, case 2 shows small respiratory motion, and case 3 is characterized by pronounced reconstruction artifacts in the moving image. In case 1, a large registration error is observed in the diaphragm region for Demons (yellow arrow). In case 3, the reconstruction artifacts are present in the original moving image and were not introduced by DIR. No specific artifact correction or masking was applied before registration. Substantial errors are observed for modified Demons and ANACONDA (purple arrows).

In the test cohort, after upsampling the DVFs to a uniform spatial resolution, the median proportion of voxels with negative Jacobian determinants $|J_{\phi }\left(p\right)|$ was below 0.002% across all methods. Figure 3 presents representative test cases with large respiratory motion, small respiratory motion, and pronounced reconstruction artifacts. In case 1, characterized by large respiratory motion, substantial errors were observed with Demons, particularly near the diaphragm. VoxelMorph also showed errors in this region. Furthermore, modified Demons, Demons, and VoxelMorph exhibited reduced registration accuracy in fine intrapulmonary vascular structures. In case 2, where respiratory motion was small, no prominent errors were observed for any methods; however, modified Demons and ANACONDA exhibited large discrepancies in body and lung contours compared with case 1. Although the deformed images produced by modified Demons appeared visually plausible, notable contour errors were consistently identified across all test cases. Case 3 involved pronounced imaging artifacts in the upper lung region and diaphragm regions, where modified Demons and ANACONDA showed reduced DIR accuracy. In contrast, Demons and VoxelMorph produced respiratory‐related deformations comparable to those obtained in artifact‐free cases.

**FIGURE 4:**
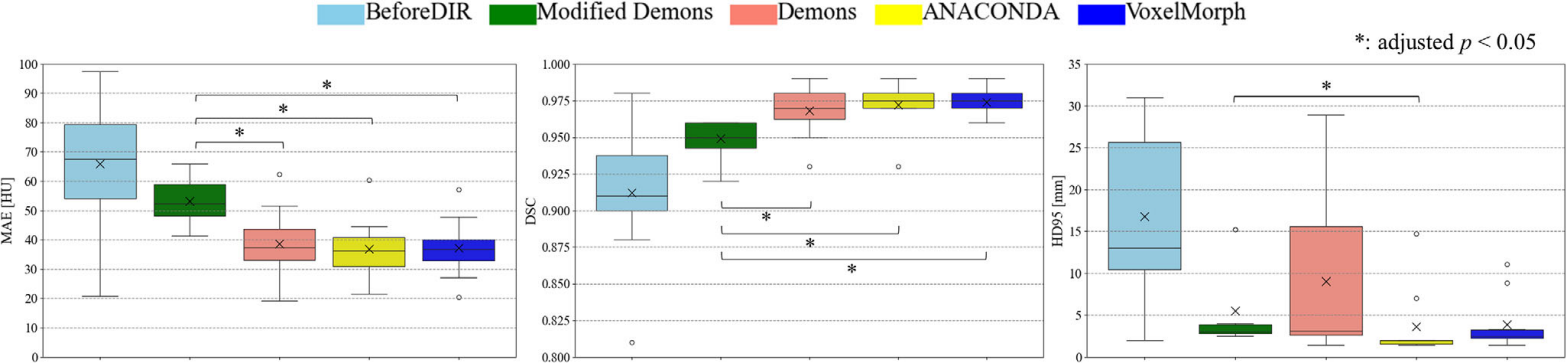
Distributions of MAE, DSC, and HD95. The left box plot shows the MAE within the body region between the deformed and fixed images. The middle and right box plots show the DSC and HD95, respectively, computed between the lung labels of the deformed and fixed images. Only statistically significant pairwise comparisons are indicated by asterisk (*).

Figure 4 shows the distributions of MAE, DSC, and HD95 across the 10 test cases. The median MAE decreased from 67.56 HU before DIR to 52.32 HU for modified Demons, 37.39 HU for Demons, 36.18 HU for ANACONDA, and 36.83 HU for VoxelMorph after DIR. The interquartile range (IQR) of MAE for VoxelMorph was 7.21 HU, indicating the smallest variability among the four methods. The median DSC increased from 0.91 before DIR to 0.95 for modified Demons, 0.97 for Demons and ANACONDA, and 0.98 for VoxelMorph. For Demons, modified Demons, and ANACONDA, cases with low pre‐DIR DSC tended to yield post‐DIR DSC values of approximately 0.92–0.93, consistent with visual assessment. In contrast, VoxelMorph achieved DSC values ≥ 0.96 even in cases with initially low DSC. The median HD95 was 3.0 mm for modified Demons, 3.1 mm for Demons, 2.0 mm for ANACONDA, and 2.5 mm for VoxelMorph. The Friedman test revealed significant differences among the four methods for three metrics (*p <* 0.05). Post hoc Wilcoxon signed‐rank tests with Holm correction revealed metric‐dependent differences among the DIR methods. Modified Demons achieved significantly higher MAE and lower DSC than Demons, ANACONDA, and VoxelMorph (adjusted *p <* 0.05), whereas no significant differences were observed among the latter three methods. For HD95, ANACONDA yielded significantly lower values than modified Demons (adjusted *p <* 0.05), while no other pairwise comparisons reached statistical significance.

The median DIR processing times (IQR) across the 10 test cases were 0.5 s (0.4–0.5 s) for VoxelMorph, 68 s (67.9–68.3 s) for Demons, 17 s (13–20 s) for modified Demons, and 34 s (14–53 s) for ANACONDA. Despite differences in computational environments and input image sizes, VoxelMorph demonstrated markedly shorter processing times with low inter‐case variability, whereas ANACONDA exhibited the largest variability.

## 4 DISCUSSION

The results of this study demonstrate that VoxelMorph achieved DIR accuracy comparable to that of the clinically used methods evaluated in this study, including modified Demons and ANACONDA. This finding is broadly consistent with DSC values previously reported in comparative studies of conventional DIR methods, such as DirOne (Cosylab d.d., Ljubljana, Slovenia), and commercial systems, including MIM Maestro (MIM Software Inc., Cleveland, OH, USA) and VelocityAI (Varian Medical Systems),^17^ supporting the potential feasibility of VoxelMorph as an alternative DIR approach in clinical practice. Although VoxelMorph achieved the highest DSC, its HD95 was slightly higher than that of ANACONDA. This difference may reflect the complementary characteristics of these metrics, as DSC primarily evaluates overall anatomical overlap, whereas HD95 is more sensitive to localized boundary discrepancies. However, as discussed below, these findings should be interpreted in light of differences in processing resolution among the evaluated methods.

In addition, VoxelMorph exhibited less inter‐case variability in both visual and quantitative evaluations than the conventional DIR methods. This characteristic may be partly attributable to differences in optimization strategies: whereas conventional DIR methods rely on case‐specific optimization, VoxelMorph applies a single trained model uniformly across cases. Consequently, DL‐based DIR approaches such as VoxelMorph may be less sensitive to case‐dependent factors, including respiratory motion magnitude and imaging artifacts, potentially enabling more stable and consistent performance across diverse clinical cases.

Despite the favorable DIR accuracy achieved by VoxelMorph, non‐physical deformations characterized by negative Jacobian determinants were not completely eliminated, reflecting the inherent trade‐off between image similarity and deformation regularity in DIR. Although the negative Jacobian determinant is useful for assessing topological consistency, it does not directly evaluate the fidelity of fine‐scale anatomical deformation. In the present study, following the approach described in Ref.,^10^ the regularization coefficient λ was systematically investigated using the validation cohort to balance these competing objectives. Stronger regularization improved deformation plausibility but tended to compromise image similarity, highlighting the importance of appropriate regularization in DL‐based DIR. Careful parameter selection is therefore essential in radiotherapy applications, in which both physically plausible and anatomically accurate deformations are required.

Importantly, VoxelMorph and Demons operated on lower‐resolution images than modified Demons and ANACONDA and may therefore have produced smoother DVFs with a limited ability to capture fine‐scale anatomical deformations. Subsequent upsampling of the VoxelMorph and Demons DVFs could not recover deformation information that was not captured during the original registration. This resolution mismatch may have increased MAE, decreased DSC, and increased HD95, particularly in regions containing fine anatomical structures. Therefore, the observed differences in these metrics may reflect not only differences among the registration algorithms but also differences in their processing resolutions. Although the low proportion of voxels with negative Jacobian determinants indicates that non‐physical folding was uncommon, this metric reflects only topological regularity and does not demonstrate an equivalent ability to capture fine‐scale anatomical deformation across the methods. The lower processing resolution may have contributed to the reduced registration accuracy observed in fine intrapulmonary vascular structures for VoxelMorph and Demons in Case 1. However, because reduced accuracy was also observed for modified Demons, which operated at near‐native resolution, differences in processing resolution alone cannot fully explain this finding.

Similarly, although VoxelMorph demonstrated substantially shorter DIR processing times than the evaluated conventional DIR algorithms, processing times were measured under different computational conditions and at different input image resolutions. Both VoxelMorph and the commercial DIR algorithms were GPU‐based; however, they differed in hardware specifications and software implementation, and VoxelMorph used lower‐resolution input images than the commercial algorithms. Therefore, the observed differences in processing time may reflect algorithmic characteristics, hardware specifications, software implementation, and input image resolution and should not be attributed solely to intrinsic algorithmic efficiency.

Nevertheless, various DL‐based DIR methods beyond VoxelMorph have been proposed,^12^, ^18^ including transformer‐based architectures such as LungRegNet, TransMorph and diffeomorphic or multiscale registration approaches.^19^, ^20^, ^21^, ^22^ These newer architectures aim to better capture long‐range spatial dependencies, preserve deformation regularity, or improve registration across multiple spatial scales, and may offer advantages over the conventional VoxelMorph framework in specific applications. VoxelMorph was nevertheless selected in the present study because it is a well‐established and widely validated DL‐based DIR framework, allowing a transparent comparison with conventional DIR methods. The comparative framework used in this study may also provide a useful basis for evaluating newer DL‐based DIR architectures in future studies. Although additional validation is required before routine clinical implementation, the present findings suggest that VoxelMorph is a promising research‐oriented DIR framework, with registration accuracy comparable to that of the evaluated conventional methods and relatively stable performance. VoxelMorph also demonstrated shorter processing times under the implementation conditions used in this study.

This study has several limitations. First, differences in image resolution and DVF upsampling procedures may have introduced bias into the comparison. Because VoxelMorph and Demons operated on lower‐resolution images than modified Demons and ANACONDA, the effects of processing resolution could not be fully separated from those of the registration algorithms. Future studies in which VoxelMorph is trained and evaluated at a spatial resolution comparable to that used by the other DIR methods are warranted to better distinguish the effect of processing resolution from that of the registration algorithm. Second, DIR accuracy was evaluated using MAE, DSC, and HD95, which assess voxel‐intensity agreement, regional overlap, and boundary alignment, respectively, but do not directly quantify pointwise anatomical correspondence, as does landmark‐based target registration error (TRE). Although TRE is widely used in benchmark datasets such as DIR‐Lab,^23^ establishing reliable corresponding landmarks in routine clinical thoracic 4D‐CT images remains challenging. Future studies incorporating landmark‐based validation in a carefully annotated subset would enable a more comprehensive assessment of DIR accuracy and strengthen the clinical relevance of the proposed framework. Finally, the training dataset was derived from a single institution, and variations in imaging protocols, respiratory motion patterns, and scanner characteristics across institutions may affect the generalizability of the proposed method. External validation using larger, multicenter datasets is therefore needed to establish the robustness and generalizability of the proposed method.

## 5 CONCLUSIONS

This study demonstrated that VoxelMorph achieves DIR accuracy comparable to clinically used algorithms for thoracic 4D‐CT in radiotherapy, together with high overlap accuracy and relatively low inter‐case variability. VoxelMorph also demonstrated shorter processing times under the implementation conditions used in this study. Although further validation under standardized spatial and computational conditions, as well as methodological refinement, is required before routine clinical implementation, VoxelMorph represents a promising research‐oriented DIR framework with potential applications in clinical radiotherapy.

## AUTHOR CONTRIBUTIONS

**Mizuha Sakai**: Conceptualization; methodology; formal analysis; investigation; data curation; writing—original draft; visualization. **Megumi Nakao**: Software; writing—review and editing. **Hideaki Hirashima**: Data curation; writing—review and editing. **Masahiro Yoneyama**: Resources; writing—review and editing. **Noriko Kishi**: Resources; writing—review and editing. **Takashi Mizowaki**: Resources; writing—review and editing. **Mitsuhiro Nakamura**: Conceptualization; writing—review and editing; visualization; supervision; project administration; funding acquisition.

## CONFLICT OF INTEREST STATEMENT

**Noriko Kishi**: Honoraria for lecture from Varian Medical Systems, and employment under a joint research agreement funded by Hitachi High‐Tech Co. ltd.; **Takashi Mizowaki**: Research grants and scholarship donations from Hitachi High‐Tech Co., Ltd.; commissioned and collaborative research projects and honoraria with Varian Medical Systems; commissioned projects and honoraria with Brainlab AG; and honoraria from Elekta and Hitachi Ltd. The other authors have nothing to disclose; **Mitsuhiro Nakamura**: Research grants and scholarship donations from Hitachi High‐Tech Co., Ltd.

## FUNDING INFORMATION

This study was supported by JSPS KAKENHI grant (Grant‐in‐Aid for Scientific Research (B) 23K24282).

## ETHICAL APPROVAL

The use of the patient data in this study was approved by the institution ethics review board (R1446‐3).

## GENERATIVE AI STATEMENT

The authors used ChatGPT (OpenAI) for language translation and for generation and modification of analysis scripts. After using this tool, the authors reviewed and edited the content as needed and take full responsibility for the content of the publication.

## Data Availability

The data are not publicly available due to privacy or ethical restrictions.
